# Landscape of interventional clinical trials involving gastrectomy for gastric cancer

**DOI:** 10.3332/ecancer.2021.1218

**Published:** 2021-04-08

**Authors:** Hussein H Khachfe, Hamza A Salhab, Mohamad Y Fares, Mohamad A Chahrour, Faek R Jamali

**Affiliations:** 1 Faculty of Medicine, American University of Beirut Medical Center, Beirut 00000, Lebanon; 2 Department of Surgery, University of Pittsburgh Medical Center, Pittsburgh, PA 15260, USA; 3 Division of GI Surgical Oncology, UPMC Pancreatic Cancer Center, University of Pittsburgh Medical Center, UPMC Cancer Pavilion, Pittsburgh, PA 15260, USA; 4 Neuroscience Research Center, Faculty of Medical Sciences, Lebanese University, Beirut 00000, Lebanon; 5 Division of General Surgery, Department of Surgery, Sheikh Shakhbout Medical City, Abu Dhabi 11001, UAE

**Keywords:** stomach, gastric cancer, gastrectomy, general surgery, clinical trials

## Abstract

**Background:**

Gastric cancer (GC) is the third most common cause of malignancy associated mortality globally. The cornerstone of curative treatment involves surgical gastrectomy. In this study, we explore clinical trials involving gastrectomy for GC, highlighting inadequacies and underlining promising surgical interventions and strategies.

**Materials and methods:**

On 1 May 2020, ClinicalTrials.gov was explored for interventional trials related to gastrectomy for GC, without adding limitations for location or date. All data pertaining to the trials were collected. Characteristics such as phase, duration, enrolment size, location, treatment allocation, masking and primary endpoint were analysed.

**Results:**

One hundred thirty-eight clinical trials met the search criteria. Clinical trials were performed in only 14 countries; most of them occurring in China. Most trials (33%) were still in the recruiting phase. On average, the length of trials was 3.9 years. Most trials had parallel assignment, were randomised and masked. The primary endpoint which was mostly commonly studied was overall survival (33%). The most common intervention studied is laparoscopic gastrectomy in 43 (31%) trials.

**Conclusions:**

Our study exposed a small number of trials, publication rate, absence of geographic variety in clinical trials involving gastrectomy for GC. Adequate management of trial design can help decrease duration and increase validity of results. More trials comparing different surgical techniques are needed to update the surgical practice of gastrectomy for GC.

## Introduction

Among all causes of death worldwide, cancers and malignancies are the second most common cause [1]. The six most prevalent cancer globally and third common cause of malignancy related mortality is gastric cancer (GC) [2]. The majority (almost 90%) of GCs are adenocarcinomas, which arise from mucosal glands of the stomach [3]. GC incidence rates vary extensively between both sexes, and across different countries and geographic locations [3]. The areas with the highest rates of GC worldwide are East Asia and Eastern Europe, while North America is home to the lowest rates in the world [4].

The cornerstone of curative treatment for this disease is surgical resection with lymphadenectomy [5]. Yet, only about 50% of all GC patients may undergo resection with curative intent. Curative resection has a ‘5-year survival rate’ of around 45%, with perioperative chemotherapy improving that rate by around 10% [6, 7].

In the past couple of decades, surgeries for GC have witnessed a shift from the traditional open approach to more minimally invasive operations [8]. These new techniques include laparoscopic assisted, total laparoscopic, robot assisted and total robotic operations. Now, minimally invasive surgical approaches have become the new standard for GC. These surgeries provide briefer hospital stay, faster recovery and general enhancement in patient quality of life [9, 10]. This shift to minimally invasive surgeries came about because of reasons such as advancements in surgical instruments, increased experience among surgeons and the better outcomes associated with them [11].

In recent years, surgery has become the primary intervention employed in the management of GC. Beneficial clinical outcomes depend heavily on finding of new surgical techniques and treatment plans. Therefore, it is vital to evaluate surgical interventions that are currently in trial or new ones that have arose. Here, we give an overview of gastrectomy clinical trials for GC, study the characteristics, discuss the inadequacies associated with them, highlight the effective interventions present and suggest potential rooms for enhancement.

## Materials and methods

### Search strategy and selection criteria

On 1 May 2020, we retrieved all information on clinical trials involving gastrectomy for GC from ClinicalTrials.gov. This search was conducted without adding limitations for date or location. ‘Clinicaltrials.gov’ is a registry that archives new data on various clinical trials weekly. For investigators to submit entries into the registry, they are required to provide extensive specifics on their particular trial. These details include trial profile, a report of protocol used in their study and any history that may be relevant. Analysis and extrapolation of conclusions on the basis of information present in this wide-ranging registry has previously been described in a number studies [12, 13].

**Figure 1.**
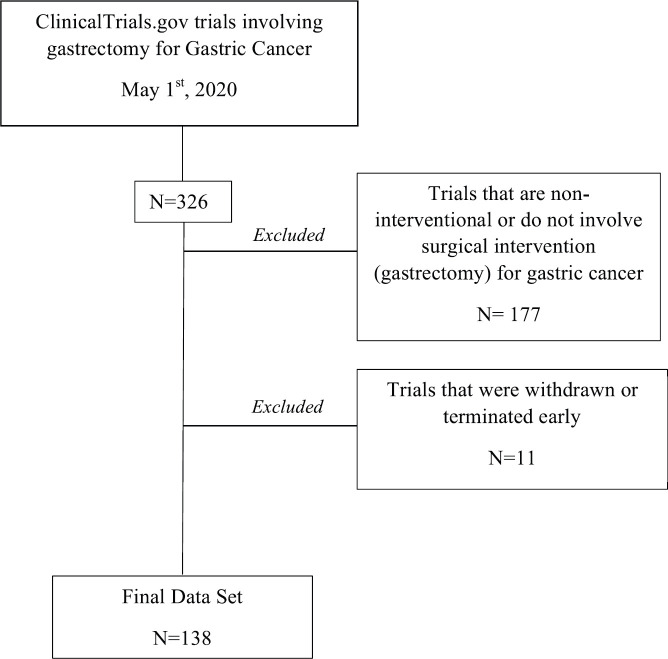
Clinical trial selection process for trials involving gastrectomy for GC from ClinicalTrials.gov.

Of the 326 total trails collected, 177 were eliminated as they were either ‘non-interventional’ or did not involve gastrectomy as an intervention. This exclusion was done using a parallel elimination plan as Nasrallah* et al* [14] where withdrawn/terminated and ‘non-interventional’ trials were not included in the final set of studies analysed (Figure 1).

### Data collection

All information relating to the clinical trials were gathered. This included: trial status (‘active not recruiting’, ‘completed’, ‘enrolling by invitation’, ‘not yet recruiting’, ‘suspended’, etc.), phase of trial (‘I’, ‘I/II’, ‘II’, ‘II/III’, ‘IV’), the official start and end/completion dates, location (city/country), selection criteria (inclusion and exclusion), primary endpoints, sample size, outcomes, interventions used and where any publications were produced. Trial duration was calculated from the official start date until the primary end/completion date. This was done to be in accordance to the Food and Drug Administration Amendments Act which was announced in 2007 (Section 801) [15]. Primary endpoints were defined as ‘30-day reoperation’, ‘number of lymph nodes harvested/collected’, ‘operation time’, ‘percentage body weight ratio’, ‘overall survival’, ‘postoperative length of stay’, ‘postoperative morbidity’, ‘postoperative outcomes’, ‘progression-free survival’, ‘rate of conversion’, ‘quality of life’, ‘time till drain removal’ and ‘tumour recurrence rate’.

### Publications produced

Articles or published manuscripts originating from trials were retrieved using the ClinicalTrials.gov identification number (NCTID) of each respective clinical trial. NCTID numbers were inserted into several search engines. The most important of which were ‘PubMed/Medline’ and ‘Scopus/Embase’. This was done to find the related published works present (if any was to be found). Should a clinical trial have had a linked published work, then the NCTID number would be included in the original publication, and the work would subsequently appear in the search. Retrieved articles/published manu scripts were gathered and subsequently reviewed by two independent authors/investigators to recognise which ones were reporting primary outcomes/results.

### Ethical approval

This study did not require/need any ethical approval of informed consent due to its epidemiologic nature (de-identified, publicly accessible data).

## Results

### Trial characteristics

**Figure 2.**
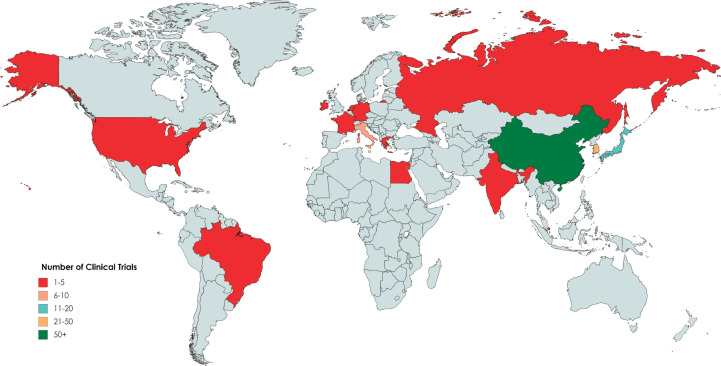
Distribution of clinical trials involving gastrectomy for GC according to ClinicalTrials.gov as of 1 May 2020.

One hundred and thirty eight trials had the criteria needed of our study. The distribution of these clinical trials was done according to characteristic details such as the number of participants, phase, status, location and duration in Table 1**.** This study showed that 39,954 participants were registered altogether in clinical trials involving gastrectomy for GC (Table 1). More than 70% of trials had >100 patients enrolled (Table 1**)**. Clinical trials were performed across only 14 different countries, with the majority taking place in Asia/Australia (Table 1, Figure 2). The duration/length was stated in all trials, with an average length of 3.9 years (Table 1). Most trials (33%) were in the recruiting phase. Almost all (95%) of the clinical trials were for adults only, and all of them (100%) were for both genders.

Trials were further assorted by interventional model, treatment allocation, masking and primary end point as in Table 2. In terms of interventional models, 82% of trials had parallel assignment (Table 2). 78% of trials were randomised and were not masked (Table 2). The most common primary endpoint was progression-free survival, where it was present in 28% of all trials in our study.

### Publications linked to trials

Of the 138 total clinical trials in our study, only 39 had linked publications related to gastrectomy surgeries for GC. A total number of 57 publications were retrieved (Table 1). Of the 33 completed trials, 28 publications were produced.

### Not applicable (NA)

Sixty-two trials (45%) were in this phase, with only 14 trials being completed (Table 1). Patients enrolled in non-applicable phase trials were 17,444. All four trials which included paediatric cases were found in this category (Table 1**)**. These trials were spread across 12 different countries, with China conducting the highest number at 39 (63%). Average trial duration in this phase was 3.3 years. Sixteen publications were linked to trials in this status (Table 1). The overwhelming majority of trials had parallel assignment (86%), were randomised (79%) and were not masked (67%) (Table 2). Progression-free survival was the most common primary endpoint in these trials with a total number of 17 (28%).

### Phase I trials

Only 2 (1%) trials were in phase I, both of which were of unknown status (Table 1). Phase I trials had 204 patients enrolled, both of which were only for adults. These trials were conducted in China and Republic of Korea (Table 1, Figure 2). Average trial length was around 3.5 years. A single publication was linked to these trials (Table 1). Both clinical trials were randomised, had parallel assignment and masked (Table 2). One trial focused on progression-free survival, while the other had a primary endpoint of quality of life (Table 2).

### Phase I/II trials

Only 3 (2%) trials were in Phase I/II, all of which were completed (Table 1). Phase I/II trials had 117 patients enrolled, all of which were only for adults. Two trials were conducted in Republic of Korea and one was done in China (Table 1, Figure 2). Average trial length was 1.8 years. Two published works were retrieved from phase I/II trials (Table 1). Single group assignment was found in two trials, while one trial had parallel assignment (Table 2). Two trials had no specified treatment allocation, while one trial was randomised. Two trials were not masked, while one trial did not specify any details on masking. The primary endpoints studied were: ‘number of harvested/collected lymph nodes’, ‘postoperative morbidity’ and ‘rate of conversion’ (Table 2).

### Phase II trials

Twenty-six (19%) trials were in phase II, only five of which were completed (Table 1). Trials of this status had 2,967 patients enrolled, all of which were adults only. Trials were distributed to six countries, with China conducting the highest number at 16 (Table 1, Figure 2). Average trial length was 2.9 years. Ten published works were retrieved from trials in this phase (Table 1). The majority of trials (54%) had parallel assignment and were randomised (Table 2). Almost all (92%) of trials had no masking (Table 2). Progression-free survival was the most common primary endpoint, which was found in seven trials (Table 2).

### Phase II/III trials

Three (2%) trials were in phase II/III, only one of which was completed (Table 1). Trials of this status had 440 patients enrolled, all of which were adults only. All trials were conducted in China (Table 1, Figure 2). Average trial length was 4.6 years. No published works were retrieved from trials in this phase. All clinical trials had parallel assignment and were masked (Table 2). Two trials were randomised, while one was non-randomised (Table 2**).** Two studies had a primary endpoint of overall survival, while one was interested in progression-free survival (Table 2).

### Phase III trials

Forty-one trials (30%) were in phase III, where 11 were completed (Table 1). Trials in this phase had 18,632 patients enrolled, all of which were adults only (Table 1). Trials were distributed to seven countries, where China had the highest number with 16 (Table 1, Figure 2). Average trial length was 5.4 years. Twenty-six publications were linked to trials in this phase (Table 1). Almost all (98%) trials had parallel assignment (Table 2), and the majority were randomised (95%) and had no masking (88%) (Table 2). The most common primary endpoint was overall survival (37%) (Table 2).

### Phase IV trials

A single (1%) trial was in phase IV, and it has an unknown status (Table 1). The trial in this phase had 150 patients enrolled, where it was for adults only. The trial was conducted in Italy (Table 1, Figure 2). Trial length was 2 years. Two publications were linked to this trial (Table 1). The trial had parallel assignment, was randomised and non-masked (Table 2). The primary endpoint was postoperative outcomes (Table 2).

### Treated topics and current research lines

The most commonly treated subtype of GC was unspecified in 78 (57%) of trials (Tables 3 and 4). This was followed by advanced GC in 47 (34%), and early GC in 13 (9%). In terms of interventions used, laparoscopic gastrectomy was most commonly studied in 43 (31%) trials (Table 3). This was followed by hyperthermic intraperitoneal chemotherapy (HIPEC) in 19 trials (14%) and robotic gastrectomy in 16 (12%) trials (Table 3).

## Discussion

### Inadequacies of clinical trials

As of May 2020, there have been 138 interventional clinical trials involving gastrectomy for GC. Surgical clinical trials for GC are rare. Of the 2,028 listed trials for GC, only 138 had experimental interventions involving gastrectomy, constituting 6.8% of the total number of trials. This low percentage and number may be accredited to several reasons.

Results from our study show that clinical trials involving gastrectomy for GC lack diversity, and that there is somewhat of a proportionality between disease burden and number of trials conducted. The overwhelming majority of trials were found in eastern Asia, which is also the most heavily burdened region in the world by the malignancy [4].

Our data shows that clinicals trials were conducted across only 14 different countries worldwide. Brazil was the only country in South America to have conducted a clinical trial on gastrectomy for GC and only Egypt had conducted a couple trials in the whole of Africa. This observation can be explained by several reasons. First, states present in less developed areas like Africa perhaps lack fiscal means and the set-up needed for research studies [16]. Globally, a great discrepancy is present in the prevalence and incidence of GC. For example, yearly age standardised incidence rates of GC in the Republic of Korea is 65.9 cases per 100,000 versus a mere 7.8 cases per 100,000 in the United States [17]. This can be explained by the notion that research on specific diseases is dependent on the burden of the disease itself. Furthermore, in a large number of countries, trials do not need to be registered in ‘ClinicalTrials.gov’. This could explain why no studies were found in many nations around the world.

On average, the length of trials in this work was 3.9 years. It may be hypothesised that the long lengths of trials are owed to schemas of the respective studies. In addition, obligatory approvals and financial/logistical backing often taken a lot of time. With better planning and optimised time managing, the lengths of studies may become shorter. This would result in faster development of new gastrectomy surgical management plans for GC. Introducing ‘new master protocols’ for the screening of patients in regard to numerous features such as race and ethnicity, genetic profile and sex could help in restructuring selection processes and in the assignment of volunteers into trials in a well-matched manner according to their profile [18]. The termination of most trials was in fact due to the slow or poor participation of patients, and discontinuation of funding. The recruitment for clinical trials has been inept to increase the number of participants [19]. Health communication strategies using advertisement and media outlets did not succeed in expanding the overall volunteer number until now [20]. However, the optimal use of electronic health records (EHR) in screening for potential candidates has proved to improve volunteer requirement into trials [21]. The recruitment of participants to gastrectomy clinical trials for GC may become more effective and quicker now that a growing number of medical centres and institutions have started to adopt EHR systems. Also, surgeons that treat patients with GC are required to stay up to date on the current gastrectomy clinical trials in order to advise said patients to volunteer in them. Previous reports shown that people are more prone to volunteer in clinical trials if their primary care physician recommends it [22]. Decreased funding has led to a substantial decrease in the number of new clinical trials and increase in the number of terminated ones [23]. Reasons such as increase in trial cost and presence of constant budgets with price inflations have caused the decreased funding of interventional clinical trials [23]. These factors can help in decreasing design and recruitment duration, prevent termination of trials and generate more viable results with larger sample sizes.

Of the 138 trials included in our study, 57 publications attributed to 39 clinical trials have been produced. This amounts to a 28% publishing rate from clinical trials involving gastrectomy for GC. Several reasons may explain this low figure. Since significant time and efforts are put into designing, setting up, conducting and analysing results of clinical trials, it may be thought that taking the decision of not publishing one made by the investigator(s) or sponsor of study. Such decisions might be taken due to discrepancies in desired versus observed results [24]. Another factor would be the decision of non-publishing bias of negative results, a phenomena that has already made its way into many clinical trials [24]. However, publishing of negative results may help other researchers in focusing future research efforts by informing others of the potential difficulties and obstacles faced during any respective trial. Publishing of said negative results could help surgeons in avoiding repetition of failing gastrectomy/gastrectomy related plans and thus divert efforts to other possible new interventions.

### Treated topics

**Figure 3.**
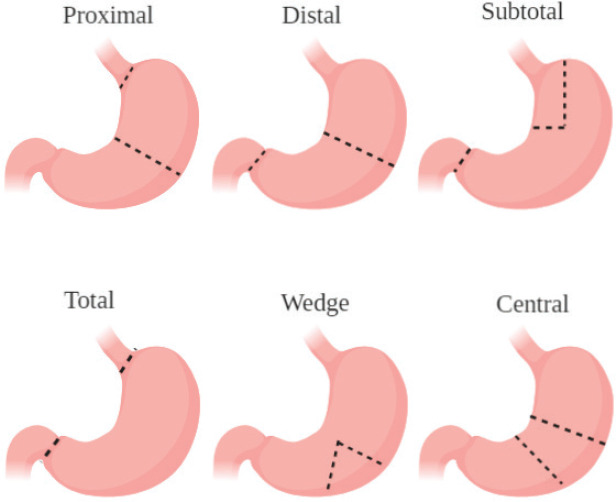
Illustration of the most common types of gastrectomies performed for GC.

The most commonly treated topics in our evaluated studies were on nonspecific GC, locally advanced gastric cancer (LAGC) and early-stage GC. This is because patients with these tumours are free from any contraindications of gastric surgery. The commonly used procedures are shown in Figure 3. The most common contraindications for gastric resection include patients being unfit for general anaesthesia and those who have extremely poor prognosis (distant metastasis) [25]. As such, clinical trials involving gastrectomy can be for these malignancies. The most commonly studied primary outcomes involved overall survival and 3-year disease-free survival (DFS) in published works from the clinical trials. Studies showed for the most commonly treated topics that different surgical approaches show no significant difference in terms of long-term survival but do help in immediate postoperative course [26, 27]. Current research lines are mainly focused on procedures including laparoscopy, HIPEC and robotic-assisted operations. This is because of the trend of utilising more and more minimally invasive procedures such as laparoscopy and robotic techniques for their better post-operative outcomes as compared to open approaches [28]. The increased use of HIPEC has also been reported in other cancers such as ovarian cancer, explaining the trend for increased trials in GC [29]. More clinical trials and publications must be produced on the common treated topics of GC (early stage and local advancement with no metastasis) to investigate ways which improve long-term outcomes. Better publishing of the current research lines and techniques being utilised is important to know their clinical outcomes.

### Limitations

The focus of this study is primarily on trials involving gastrectomy for patients with GC only. The strength of the results generated depends on the accuracy of the information from the source database (‘ClinicalTrials.gov’). Our study utilises the ClinicalTrials.gov database alone. The use of other databases such as the WHO International Clinical Trials Registry Platform or Cochrane database for clinical trials could have generated possibly more clinical trials. Inaccuracies might have been present in the trial data such as whether the data is up-to-date or not. Other issues could have been that data might be missing altogether from the registry.

## Conclusion

Clinical trials involving gastrectomy for GC have a small number, minimal publishing rate and lack of geographic variety. Laparoscopy is the most common intervention being studied in clinical trials involving gastrectomy. Increased research efforts, funding and proper management are needed to improve and expand clinical trials, which in turn will improve patient outcomes.

## List of abbreviations

ERAS, Enhanced recovery after surgery; LAGC, Locally advanced gastric cancer; UAS, Ultrasonically activated shears; EBL, Estimated blood loss; LDG, Laparoscopic distal gastrectomy; ODG, Open distal gastrectomy; DFS, Disease-free survival.

## Funding

The authors declare that this study did not receive any funding.

## Conflicts of interest

The authors declare no conflict of interest.

## Compliance with ethical standards

The authors declare no conflict of interest. This study did not include any research involving human participants and/or animal subjects.

## Authors’ contributions

H.H.K and M.A.C were responsible for the concept and design of the study; H.H.K and H.A.S for data acquisition; H.H.K and M.Y.F for statistical analysis; H.H.K, H.A.S, M.Y.F and F.R.J interpreted the results; H.H.K, M.A.C and F.R.J analysed the data and drafted the manuscript. All authors critically revised the manuscript, approved the final version to be published and agree to be accountable for all aspects of the work.

## Figures and Tables

**Table 1.** Characteristics of trials involving gastrectomy for GC as found on ClinicalTrials.gov as of 1 May 2020.

	NA	Phase I	Phase I/II	Phase II	Phase II/III	Phase III	Phase IV	Total (%)
Number of trials	62	2	3	26	3	41	1	138 (100%)
**Trial status**								
Active, not recruiting	6	-	-	2	-	1	-	9 (7%)
Completed	14	-	3	5	-	11	-	33 (24%)
Enrolling by invitation	4	-	-	1	-	1	-	6 (4%)
Not yet recruiting	4	-	-	-	-	2	-	6 (4%)
Recruiting	23	-	-	7	2	14	-	46 (33%)
Unknown status	11	2	-	11	1	12	1	38 (28%)
**Estimated enrolment**								
0–10	1	-	-	-	-	-	-	1 (1%)
11–50	9	-	2	4	-	3	-	18 (13%)
51–100	8	1	1	6	-	2	-	18 (13%)
>100	44	1	-	16	3	36	1	101 (73%)
Results present	-	-	-	1	-	-	-	1 (1%)
Publication	16	1	2	10	-	26	2	57 (41%)
Age group								
Adult only	55	2	3	26	3	41	1	131 (95%)
Adult and paediatric	7	-	-	-	-	-	-	7 (5%)
Paediatric only	-	-	-	-	-	-	-	0
**Gender**								
Both	62	2	3	26	3	41	1	138 (100%)
Male	-	-	-	-	-	-	-	0
Female	-	-	-	-	-	-	-	0
**Trial location**								
Americas	1	-	-	2	-	-	-	3 (2%)
Europe/UK/Russia	7	-	-	2	-	5	1	15 (11%)
Asia/Australia	52	2	3	22	3	36	-	118 (86%)
Africa	2	-	-	-	-	-	-	2 (1%)
**Trial duration (years)**								
<1	3	-	2	4	-	1	-	10 (7%)
1–5	50	2	1	21	2	24	1	101 (73%)
5–10	9	-	-	1	1	12	-	23 (17%)
10+	-	-	-	-	-	4	-	4 (3%)

**Table 2.** Study design and primary endpoints of clinical trials involving gastrectomy for GC as found on ClinicalTrials.gov as of 1 May 2020.

	NA	Phase I	Phase I/II	Phase II	Phase II/III	Phase III	Phase IV	Total (%)
**Interventional mode**								
Single group assignment	10	-	2	12	-	1	-	25 (18%)
Parallel assignment	52	2	1	14	3	40	1	113 (82%)
**Treatment allocation**								
Nonrandomised	6	-	-	-	1	1	-	8 (6%)
Randomised	49	2	1	14	2	39	1	108 (78%)
Not specified	7	-	2	12	-	1	-	22 (16%)
**Masking**								
Open label (none)	42	-	2	24	3	36	-	107 (78%)
Masked	20	2	-	2	-	5	1	30 (22%)
Not specified	-	-	1	-	-	-	-	1 (1%)
**Main primary endpoint**								
30-day reoperation	1	-	-	-	-	-	-	1 (1%)
Number of harvested lymph nodes	5	-	1	5	-	3	-	14 (10%)
Operation time	4	-	-	1	-	1	-	6 (4%)
Overall survival	4	-	-	4	2	15	-	25 (18%)
Percentage body weight ratio	-	-	-	-	-	1	-	1 (1%)
Postoperative length of stay	3	-	-	1	-	1	-	5 (4%)
Postoperative morbidity	6	-	1	2	-	2	-	11 (8%)
Postoperative outcomes	16	-	-	5	-	3	1	25 (18%)
Progression-free survival	17	1	-	7	1	12	-	38 (28%)
Quality of life	3	1	-	1	-	3	-	8 (6%)
Rate of conversion	-	-	1	-	-	-	-	1 (1%)
Time to drain removal	1	-	-	-	-	-	-	1 (1%)
Tumour recurrence rate	2	-	-	-	-	-	-	2 (1%)

**Table 3.** Distribution of treated topics and interventions used in clinical trials involving GC.

Treated topic	Number of trials (%)
Early GC	13 (9%)
Advanced GC	47 (34%)
GC (unspecified stage)	78 (57%)
**Intervention used**	
Intracorporeal oesophagojejunostomy	1 (1%)
Vagus nerve-preservation	2 (1%)
Robotic gastrectomy	16 (12%)
Laparoscopic gastrectomy	43 (31%)
HIPEC	19 (14%)
Endoscopic submucosal dissection	1 (1%)
Carbon nanoparticles	1 (1%)
Standardised 400 kcal meal	1 (1%)
Double tract reconstruction	1 (1%)
Enhanced recovery after surgery programme	5 (4%0
Ultrasonic activated shears (UAS)	2 (1%)
Open gastrectomy	8 (6%)
Billroth reconstruction	6 (4%)
Perianastomotic drain	4 (3%)
Lymphadenectomy	9 (7%)
Application of third space	1 (1%)
Prophylactic cholecystectomy	1 (1%)
Nasogastric decompression	1 (1%)
Spleen-preservation	2 (1%)
Laparoscopic enforced sutures	1 (1%)
Adjuvant chemotherapy	6 (4%)
Neoadjuvant chemotherapy	3 (2%)
Roux-en-Y reconstruction	2 (1%)
Perioperative electropuncture	1 (1%)
Total omentectomy	1 (1%)

**Table 4.** Clinical findings of interventional clinical trials involving gastrectomy for GC.

Authors	Year	Trial	NCTID	Number enrolled	Inclusion criteria	Primary outcome	Result
Sakuramoto *et al* [30]	2007	Adjuvant chemotherapy for gastric cancer with S-1, an oral fluoropyrimidine	NCT00152217	529	LAGC	Overall survival	Oral fluoropyrimidine is an effective adjuvant treatment for LAGC
Nakajima *et al* [31]	2007	Randomized controlled trial of adjuvant uracil-tegafur versus surgery alone for serosa-negative, locally advanced gastric cancer	NCT00152243	190	Seronegative, node positive GC	Overall survival	Significant survival benefit for postoperative adjuvant chemotherapy with uracil-tegafur
Sasako *et al* [32]	2008	D2 lymphadenectomy alone or with para-aortic nodal dissection for gastric cancer	NCT00149279	523	GC	Overall survival	Treatment with D2 lymphadenectomy plus para-aortic nodal dissection does not improve the survival rate
Iwahashi *et al* [27]	2009	Evaluation of double tract reconstruction after total gastrectomy in patients with gastric cancer: prospective randomized controlled trial	NCT00746161	44	GC	Quality of life	No difference between double tract and Roux-En-Y for total gastrectomy
Miyashiro *et al* [33]	2011	Randomized clinical trial of adjuvant chemotherapy with intraperitoneal and intravenous cisplatin followed by oral fluorouracil (UFT) in serosa-positive gastric cancer versus curative resection alone: final results of the Japan Clinical Oncology Group trial JCOG9206-2	NCT00147147	268	GC	Overall survival	No benefit in overall and relapse-free survival with intraperitoneal cisplatin, postoperative intravenous cisplatin and 5-FU
Kim *et al* [26]	2013	Long-term outcomes of laparoscopy-assisted distal gastrectomy for early gastric cancer: result of a randomized controlled trial (COACT 0301)	NCT00546468	164	Early distal GC	5-year DFS	No difference in long-term benefits between laparoscopic distal gastrectomy (LDG) and open distal gastrectomy (ODG)
Lee *et al* [34]	2013	Morbidity and mortality after laparoscopic gastrectomy for advanced gastric cancer: results of a phase II clinical trial	NCT01441336	204	LAGC	Feasibility of laparoscopic gastrectomy	LG with D2 lymphadenectomy is safe and feasible
Bernini *et al* [35]	2013	The Cholegas Study: safety of prophylactic cholecystectomy during gastrectomy for cancer: preliminary results of a multicentric randomized clinical trial	NCT00757640	172	GC	Evaluation of the incidence of cholelithiasis postoperatively	Concomitant cholecystectomy adds no extra perioperative morbidity, mortality and costs
Haverkamp *et al* [5]	2015	Laparoscopic versus open gastrectomy for gastric cancer, a multicenter prospectively randomized controlled trial (LOGICA-trial)	NCT02248519	210	Surgically resectable GC	Postoperative hospital stay	Laparoscopic surgery provides shorter hospital stay
Abdikarim *et al* [36]	2015	Enhanced recovery after surgery with laparoscopic radical gastrectomy for stomach carcinomas	NCT01955096	61	GC	Postoperative hospital stay	ERAS programme is associated with shorter hospital stay
Nakamura *et al* [37]	2016	Randomized clinical trial comparing long-term quality of life for Billroth I versus Roux-en-Y reconstruction after distal gastrectomy for gastric cancer	NCT01065688	122	GC	Quality of life	No difference between Billroth I and Roux-en-Y reconstruction
Oh *et al* [38]	2017	Ultrasonically Activated Shears Reduce Blood Loss without Increasing Inflammatory Reactions in Open Distal Gastrectomy for Cancer: A Randomized Controlled Study	NCT01971775	56	GC	Estimated blood loss (EBL) during surgery	UAS reduced EBL without increasing inflammatory reactions
Lee *et al* [39]	2017	Safety and feasibility of reduced-port robotic distal gastrectomy for gastric cancer: a phase I/II clinical trial	NCT02347956	40	Early GC	30-day morbidity and mortality	Reduced-port robotic distal gastrectomy could be a valid alternative to conventional robot distal gastrectomy
Park *et al* [40]	2018	Laparoscopy-Assisted versus Open D2 Distal Gastrectomy for Advanced Gastric Cancer: Results From a Randomized Phase II Multicenter Clinical Trial (COACT 1001)	NCT01088204	204	LAGC	Noncompliance rate of lymph node dissection	LDG is feasible for D2 lymph node dissection
Kang *et al* [41]	2018	Multimodal Enhanced Recovery After Surgery (ERAS) Program is the Optimal Perioperative Care in Patients Undergoing Totally Laparoscopic Distal Gastrectomy for Gastric Cancer: A Prospective, Randomized, Clinical Trial	NCT01938313	97	GC	Recovery time	ERAS is safe and enhances postoperative recovery after total laparoscopic distal gastrectomy in GC
Zheng *et al* [42]	2018	Comparison of 3D laparoscopic gastrectomy with a 2D procedure for gastric cancer: A phase 3 randomized controlled trial	NCT02327481	438	GC	Short-term postoperative complications and mortality	3D laparoscopic gastrectomy does not shorten the operation time compared with 2D laparoscopic gastrectomy, but provides less intraoperative blood loss and a lesser occurrence of excessive bleeding
Li *et al* [43]	2019	Assessment of Laparoscopic Distal Gastrectomy After Neoadjuvant Chemotherapy for Locally Advanced Gastric Cancer: A Randomized Clinical Trial	NCT02404753	96	LAGC receiving neoadjuvant therapy	3-year recurrence free survival	LDG provides better outcomes than the ODG approach
Ahn *et al* [44]	2019	Long-term Survival Outcomes of Laparoscopic Gastrectomy for Advanced Gastric Cancer: Five-year Results of a Phase II Prospective Clinical Trial	NCT01441336	157	LAGC	3-year recurrence free survival	Laparoscopic gastrectomy with D2 lymphadenectomy shows acceptable 3-year DFS
Yu *et al* [45]	2019	Effect of Laparoscopic vs Open Distal Gastrectomy on 3-Year Disease-Free Survival in Patients With Locally Advanced Gastric Cancer: The CLASS-01 Randomized Clinical Trial	NCT01609309	1056	LAGC	3-year recurrence free survival	LDG is not significantly superior to ODG
Kim *et al* [46]	2019	Effect of Laparoscopic Distal Gastrectomy vs Open Distal Gastrectomy on Long-term Survival Among Patients With Stage I Gastric Cancer: The KLASS-01 Randomized Clinical Trial	NCT00452751	1416	Stage I GC	5-year DFS	LDG is a safe alternative to ODG for stage I GC
Guo *et al* [47]	2019	Combined Surgery and Extensive Intraoperative Peritoneal Lavage vs Surgery Alone for Treatment of Locally Advanced Gastric Cancer: The SEIPLUS Randomized Clinical Trial	NCT02745509	662	LAGC	Short-term postoperative complications and mortality	Patients with LAGC appear to be candidates for the extensive intraoperative peritoneal lavage approach
Wang *et al* [48]	2019	Short-term surgical outcomes of laparoscopy-assisted versus open D2 distal gastrectomy for locally advanced gastric cancer in North China: a multicenter randomized controlled trial	NCT02464215	446	LAGC	Morbidity and mortality within 30 postoperative days	LDG was safe and feasible compared with conventional ODG
Chen *et al* [49]	2020	Safety and Efficacy of Indocyanine Green Tracer-Guided Lymph Node Dissection During Laparoscopic Radical Gastrectomy in Patients With Gastric Cancer: A Randomized Clinical Trial	NCT03050879	266	Potentially resectable GC	Number of retrieved lymph nodes	Indocyanine green improve the number of lymph node dissections and reduce lymph node noncompliance without increased complications
